# CHANGE IN EPISODIC MEMORY AND EXECUTIVE FUNCTIONING PREDICTS MORTALITY RISK

**DOI:** 10.1093/geroni/igac059.342

**Published:** 2022-12-20

**Authors:** Kaleena Odd, Julie Boron, Jacob Alderson, Margie Lachman, Nicholas Turiano

**Affiliations:** University of Nebraska Omaha, Omaha, Nebraska, United States; University of Nebraska, Omaha, Nebraska, United States; West Virginia University, Morgantown, West Virginia, United States; Brandeis University, Waltham, Massachusetts, United States; West Virginia University, Morgantown, West Virginia, United States

## Abstract

The current study explored whether the Brief Test of Adult Cognition via Telephone (BTACT) assessment could be used to predict longevity in a national sample of adults from the Midlife Development in the United States (MIDUS) study. Specifically, we examined whether 9-year changes in episodic memory (EM) and executive functioning (EF) predicted all-cause mortality risk (2004-2018). The sample included 2,643 participants (MAge=45.87; 92.23% white; 107 deceased) who completed the BTACT twice: between 2004-06 and between 2013-2017. Using change scores, decreases in EM (HR= 1.45 [1.09-1.93], p=.01) and EF (HR=1.585 [1.17-2.14], p<.001) increased the hazard of dying (controlling for age, gender, race, education, and self-rated health). Results suggest the BTACT is sensitive enough to detect health-consequential decreases in EM/EF. Future research should consider the BTACT as a viable assessment tool for older adults who may not have easy access to cognitive screenings.

